# Hepatitis A virus infections and outbreaks in asylum seekers arriving to Germany, September 2015 to March 2016

**DOI:** 10.1038/emi.2017.11

**Published:** 2017-04-26

**Authors:** Kai Michaelis, Jürgen J Wenzel, Klaus Stark, Mirko Faber

**Affiliations:** 1 Division of Gastrointestinal Infections, Zoonoses and Tropical Infections, Department for Infectious Disease Epidemiology, Robert Koch Institute (RKI), D-13353 Berlin, Germany; 2 National Consultant Laboratory for HAV and HEV, Institute of Clinical Microbiology and Hygiene, University Medical Centre Regensburg, D-93053 Regensburg, Germany

**Keywords:** asylum seeker, epidemiology, hepatitis A, hepatitis A virus, migration, refugee

## Abstract

From September 2015 to March 2016, hepatitis A notifications in Germany increased by 45% to 699 cases compared to 482 cases in the same period of the previous year. Children aged five to nine years were predominantly affected (22% of all cases in this period). We hypothesized that this increase could be explained by the marked influx of asylum seekers in this time period. We analysed national surveillance data and estimated the number of imported and autochthonous hepatitis A cases in asylum seekers. We also investigated molecular signatures of hepatitis A viruses sampled from asylum seekers to identify chains of transmission. We found that 40% (278 cases) of all 699 hepatitis A cases notified between September 2015 and March 2016 in Germany concerned asylum seekers. Most infections were acquired abroad, but at least 24% accounted for autochthonous infections. Among asylum seekers, children aged five to nine years were overrepresented with 97 of 278 (35%) notified cases. The analysed hepatitis A virus sequences were primarily subgenotype IB strains and clustered with previously isolated samples from the Middle East, Turkey, Pakistan and East Africa. Except one transmission from an asymptomatic child to a nursery nurse working in a mass accommodation, we are not aware of infection chains involving asylum seekers and German residents. We conclude that asylum-seeking children and adolescents are susceptible to hepatitis A virus infections, particularly children aged five to nine years. Measures to prevent secondary infections in asylum seekers such as extended hygiene measures and post-exposure prophylaxis seem advisable.

## INTRODUCTION

In 2015, ongoing conflicts in Africa and Asia, particularly in the Middle East led to a large influx of asylum seekers into the EU, culminating in the second half of the year.^1^ By the end of 2015, Germany hosted more than one million new asylum seekers most of them fleeing from those conflict regions.^2^ Between September 2015 and March 2016, parallel to peaking numbers of asylum seekers arriving in Germany, notified cases of hepatitis A in Germany increased substantially. Thereafter case counts returned to levels of preceding years.

Hepatitis A virus (HAV) infections occur in people of all ages. The virus is the causative agent of hepatitis A, a viral inflammatory disease that affects the liver. The severity of the disease ranges from a mild illness lasting a few weeks to a severe illness lasting several months.^3^ Symptomatic disease is associated with nausea, fever, abdominal pain, fatigue and typical signs of jaundice. In general, symptoms are more severe in the elderly and in people with weakened immune systems.^4^ Patients with chronic liver diseases are at risk to develop fulminant hepatitis A with a possible fatal outcome.^5^ In young children, hepatitis A is often asymptomatic. The period of maximum infectiveness spans one to two weeks before symptom onset until a few days after the onset of jaundice.

HAV is typically transmitted via the faecal–oral route. The virus particles are extraordinarily robust and generally stable at high temperatures, very low pH values or other harsh environmental conditions.^6^ Living conditions such as poor hygiene, crowded housing, shortage of clean drinking water or consumption of contaminated food are well-established factors, known to trigger the spread of HAV.^7^ As many of the asylum seekers experienced at least an episode of serious living conditions during the journey from their country of origin to the host country in Europe, they are potentially exposed to HAV.^8, 9^

In our report, we analyse the increase in hepatitis A cases notified in Germany between September 2015 and March 2016. Specifically, we investigate HAV transmission within the group of asylum seekers after arrival to Germany and the potential of transmission into the residential population. To this end, we assess autochthonous HAV transmission among asylum seekers and analyse transmission chains by molecular surveillance. With our report, we aim to guide public health measures mainly in regard to arriving asylum seekers but also for the general population in order to prevent further secondary transmission events of HAV.

## MATERIALS AND METHODS

### Main investigation

In Germany, all hepatitis A cases are notifiable to local health departments irrespective of nationality or status of residence of the case. Local health departments are responsible for case investigations and prevention measures. Individual case data are compiled in a case report, anonymized and electronically transmitted to the state health department and further to the Robert Koch Institute (RKI).^10, 11, 12^ We analysed the German national surveillance data for the period from September 2015 to March 2016 and ascertained imported and autochthonous hepatitis A cases in asylum seekers. Furthermore, we investigated frequency and sizes of hepatitis A outbreaks related to arriving asylum seekers. In addition, to examine possible chains of HAV transmission between asylum seekers and to confirm clusters in mass accommodations, we performed molecular typing in cooperation with the national consultant laboratory for HAV (University Medical Centre Regensburg, Germany).

### Definition of the term asylum seeker

The term asylum seeker was used in order to describe individuals that—for any reason—applied for asylum or had the intention to apply for asylum in Germany. In the investigated period, between September 2015 and March 2016, these individuals were mainly, but not exclusively refugees fleeing from conflict regions in the Middle East or East Africa.

### Age definitions

In our study, children refers to individuals aged 0–12 years, teenagers 12–19 years, adolescents 13–17 years and adults >18 years, respectively.

### Collection of case data and material for molecular surveillance

The German Protection against Infection Act (IfSG) authorizes local health departments to investigate the context of cases and to arrange appropriate prevention measures. To this end, there are no predefined standard operation procedures. However, cases are usually contacted and interviewed by a local health officer. After anonymization of patient data, gathered information is categorized in predefined reporting categories and recorded in the national surveillance data base.

For this report, we specifically analysed demographic variables introduced into the surveillance system in September 2015, allowing a differentiation of residents and asylum seekers. This recent addition to the surveillance system was made in reaction to the strong influx of asylum seekers in the second half of 2015 to provide data on the impact of this population increase on the surveillance data.

For investigation of molecular clusters, sampling is not mandatory in Germany. However, local health departments are encouraged to cooperate with the national consultant laboratory for secondhand use of collected clinical material or, occasionally, sample additional material from cases (for example, stool) for molecular analyses.

### Case definition

A case of hepatitis A was defined as an individual with one of the following clinical symptoms indicative of acute hepatitis: fever, abdominal pain, icterus, elevated transaminases and additionally a laboratory confirmation of a present HAV infection (typically based on IgM or PCR) or alternatively with an epidemiological link to a laboratory-confirmed case. Asymptomatic cases, not meeting the predefined case definition, were not considered in this study.

### Statistical data on asylum seekers

Baseline statistics of arriving asylum seekers, for example, counts, country of origin and age distribution were adopted from published documents of the Federal Office for Migration and Refugees or requested from there.^13^ Asylum seekers primarily originated from regions where HAV is endemic, Syria (50.9%), Iraq (11.1%), Afghanistan (9.5%), Albania (5.2%), Eritrea (1.8%), Pakistan (1.8%) and Serbia (1.3%). According to the WHO criteria, the level of HAV endemicity by country of origin was mainly intermediate, except for asylum seekers from East Africa (high) and from the Western Balkan countries (intermediate to low).

### Classification of imported and autochthonous cases

The incubation period of hepatitis A is 30 days on average and ranges from 15 to 50 days.^14^ In order to quantify the proportion of imported vs. autochthonous hepatitis A cases, we calculated the number of days between arrival in Germany and the reported onset of symptoms and analysed the respective distribution. We classified cases into imported (onset <15 days post arrival in Germany), probably imported (15–29 days), probably autochthonous (30–50 days) and autochthonous (>50 days) cases.

### Definition of hepatitis A outbreaks

A hepatitis A outbreak is the occurrence of a cluster with two or more cases of hepatitis A with an epidemiological link (for example, a confirmed or assumed identical source of infection).

### Statistical analysis

All hepatitis A cases notified between September 2015 and March 2016 were extracted from the national surveillance database Surv*N*et and transferred to Microsoft Excel for data management. We employed GraphPad Prism for basic statistical procedures and graphical presentation. When comparing multiple groups and Gaussian distribution was not assumed, data were analysed by Kruskal–Wallis test; a value of *P*<0.05 was considered to be statistically significant.

### Molecular typing of the VP1/P2A region of HAV samples

Molecular analyses were performed at the national consultant laboratory for HAV. In order to enable cluster analysis using molecular methods, local health departments were asked to provide blood samples of sporadic and outbreak cases of hepatitis A for molecular typing. Nucleic acid isolation was performed using the RNeasy Mini Kit (Qiagen, Hilden, Germany) with 100 μL elution volume (RNase-free water). A 10 μL aliquot of the eluate was used for reverse transcription in a total reaction volume of 20 μL using Moloney murine leukaemia virus (M-MuLV) reverse transcriptase (Applied Biosystems, Foster City, CA, USA) and random hexamers according to the manufacturer's instructions (42 °C, 30 min). Two replicates were analysed in 30 μL PCRs each containing 10 μL of the RT product (corresponding to 5 μL eluate), ROX buffer, 5.0 mmol/L MgCl_2_, 1.25 U AmpliTaq Gold DNA polymerase (all Applied Biosystems), dNTPs, specific primers (300 nmol/L each) and TaqMan hydrolysis probe (200 nmol/L). Primers and probe for the HAV reverse transcription quantitative real-time PCR (RT-qPCR) assay were in the viral polymerase gene region (SH-Poly-A, SH-Poly-1 and SH-Poly-Q^15^). Thermal cycling was performed on a StepOnePlus instrument (Applied Biosystems) and comprised a 10-min initial enzyme activation step at 95 °C, and 45 cycles of 95 °C for 15 s and 60 °C for 1 min.

RT-qPCR-positive samples were further characterized by amplicon sequencing. The amplification was performed according to the unified HAV Net protocol (http://www.rivm.nl/en/Topics/H/HAVNET) by using specific primers for the HAV VP1/P2A genomic region (HAV 6.1, 5′-TAT GCY ITI TCW GGI GCI YTR GAY GG-3′ HAV 10, 5′-TCY TTC ATY TCW GTC CAY TTY TCA TCA TT-3′, 614 nt). A 2.5 μL aliquot from the first round of PCR was then used as a template in the second round of PCR with primers HAV 8.2, 5′-GGA TTG GTT TCC ATT CAR ATT GCN AAY TA-3′ and HAV 11, 5′-CTG CCA GTC AGA ACT CCR GCW TCC ATY TC-3′ (518 nt). The PCR products were purified by using QIAquick columns (Qiagen) and sequenced in both directions with the second-round amplification primers.

Nucleotide sequences of amplicons were determined by using the BigDye Terminator Cycle Sequencing Kit (Applied Biosystems) and separated on a model 3730xl genetic analyser (Applied Biosystems). Sequences were analysed by using CodonCode Aligner 5.1.5 software (CodonCode Corporation, Centerville, MA, USA).

GenBank and local sequence databases were searched for sequences with high similarity using the BLAST and FASTA algorithms. A rooted maximum likelihood phylogenetic consensus tree for VP1/P2A nucleotide sequences was inferred using RAxML 8.2.7 software (http://sco.h-its.org/exelixis/web/software/raxml).

## RESULTS

### Notified hepatitis A cases from September 2015 to March 2016 in Germany

From September 2015 to March 2016 hepatitis A notifications in Germany rose by 45% (217 cases) to 699 cases compared to 482 cases in the same period in 2014/2015 (median of the previous five notification periods September to March: 493 cases). Of all 699 hepatitis A cases notified between September 2015 and March 2016, male cases were overrepresented with a proportion of 59%. Regarding age, children aged five to nine years were predominantly affected and accounted for 22% of the 699 cases notified in this period.

### Hepatitis A incidence in asylum seekers

**Figure 1:**
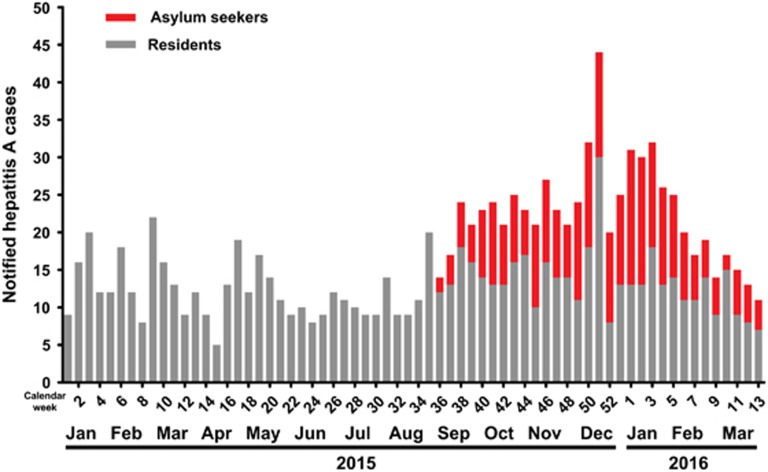
Epidemic curve of notified hepatitis A cases by week and year of notification, Germany, January 2015 through March 2016 (*n*=1126).

In total, 278 cases of hepatitis A in asylum seekers were reported between September 2015 and March 2016, and accounted for 40% of the 699 notified hepatitis A cases in Germany (Figure 1). In this period, hepatitis A was among the five most frequently notifiable infectious diseases in Germany for asylum seekers. The remaining 421 cases out of the 699 hepatitis A cases notified from September 2015 to March 2016 in Germany were cases within the residential population, but presumably included an unknown number of cases being unclassified asylum seekers (due to incomplete notifications of asylum status). From September 2015 to March 2016, a total of 852 066 arriving asylum seekers were officially recorded by the Federal Office for Migration and Refugees in Germany. Using this official figure as the denominator of the arriving population, we calculated the reporting incidence of hepatitis A in asylum seekers as compared to the residential population. The German residential population totalled 81.8 million residents in 2015. With 278 hepatitis A cases occurring per 852 066 asylum seekers and 421 cases per 81.8 million German residents, the reporting incidence of hepatitis A amounted to 32 per 100 000 and 0.5 per 100 000, respectively (reporting incidence ratio (95% confidence interval (95% CI)): 63 (54–73; *P*<0.0001).

### Imported versus autochthonous hepatitis A cases in asylum seekers

**Figure 2:**
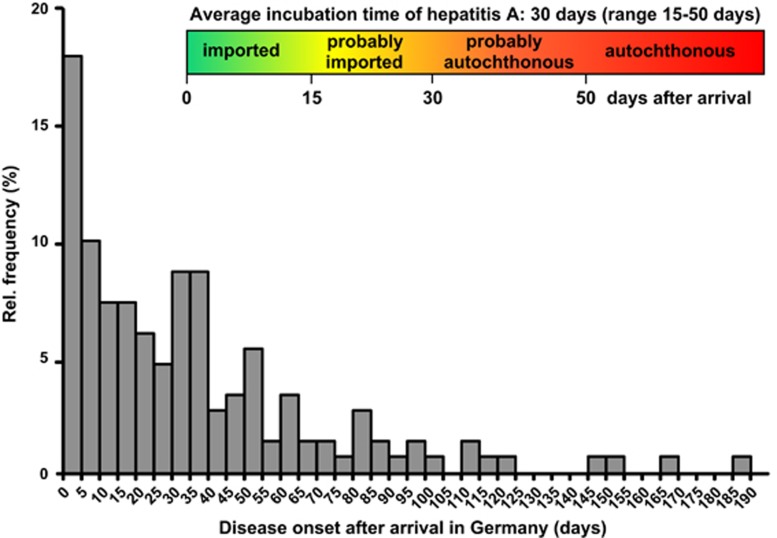
Histogram of reported disease onset of hepatitis A relative to arrival in Germany (*n*=153 cases of asylum seekers).

Both arrival and disease onset dates were available for a total of 153 (55%) hepatitis A cases in asylum seekers notified from September 2015 to March 2016 in Germany. The respective distribution of the reported times-to-disease onset after arrival is depicted in Figure 2. Time-to-disease onset after arrival ranged from 0 to 192 days. Most hepatitis A cases in asylum seekers occurred within a few days after arrival to Germany. Correspondingly, the majority (53%) of arriving asylum seekers was infected before arrival in Germany, as denoted by time-to-disease onset after arrival of <30 days and the related 53^rd^ percentile of the analysed distribution. The remaining 47% were autochthonous hepatitis A cases with HAV infections acquired after arrival in Germany. The 53% imported hepatitis A cases split into 36% of clearly imported cases (disease onset <15 days after arrival) and 17% of probably imported cases (15–30 days after arrival). The 47% autochthonous hepatitis A cases split into 24% of clearly autochthonous cases (disease onset >50 days after arrival) and 23% of probably autochthonous cases (30–50 days after arrival).

### Age distribution of hepatitis A cases from September 2015 to March 2016 in Germany

**Figure 3:**
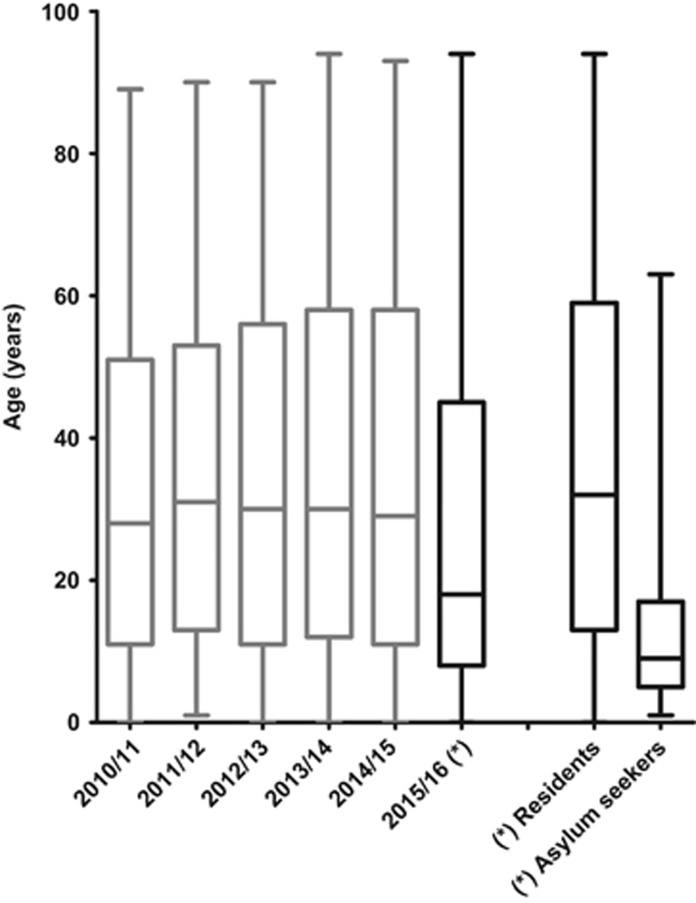
Age distribution of notified hepatitis A cases by September to March periods, Germany, 2010–2016 and age distribution of notified hepatitis A cases stratified in residents and in asylum seekers (September 2015–March 2016). Stratification and related subgroups are indicated (asterisks).

Median age of all 699 hepatitis A cases notified between September 2015 and March 2016 in Germany dropped markedly to 18 years (interquartile range (IQR): 8–45 years) as compared to the median age of all 482 hepatitis A cases notified 1 year before (29 years, IQR: 11–58 years). In the five preceding periods from September to March in 2010/2011 to 2014/2015 median age was 30 years (IQR: 11–55 years) and the age distribution was largely similar (Figure 3).

The median age of the 278 asylum seekers out of the 699 hepatitis A cases notified between September 2015 and March 2016 was nine years (IQR: 5–17 years) and the median age of the remaining 421 hepatitis A cases in German residents was 32 years (IQR: 13–59 years).

Children or teenager aged 0–19 years accounted to 53% (373 cases) of all 699 hepatitis A cases notified from September 2015 to March 2016. The proportion of children aged 5–9 years was 22% (155 cases) of all 699 cases notified in this period.

### Age distributions of arriving asylum seekers and of hepatitis A cases in this group

**Figure 4:**
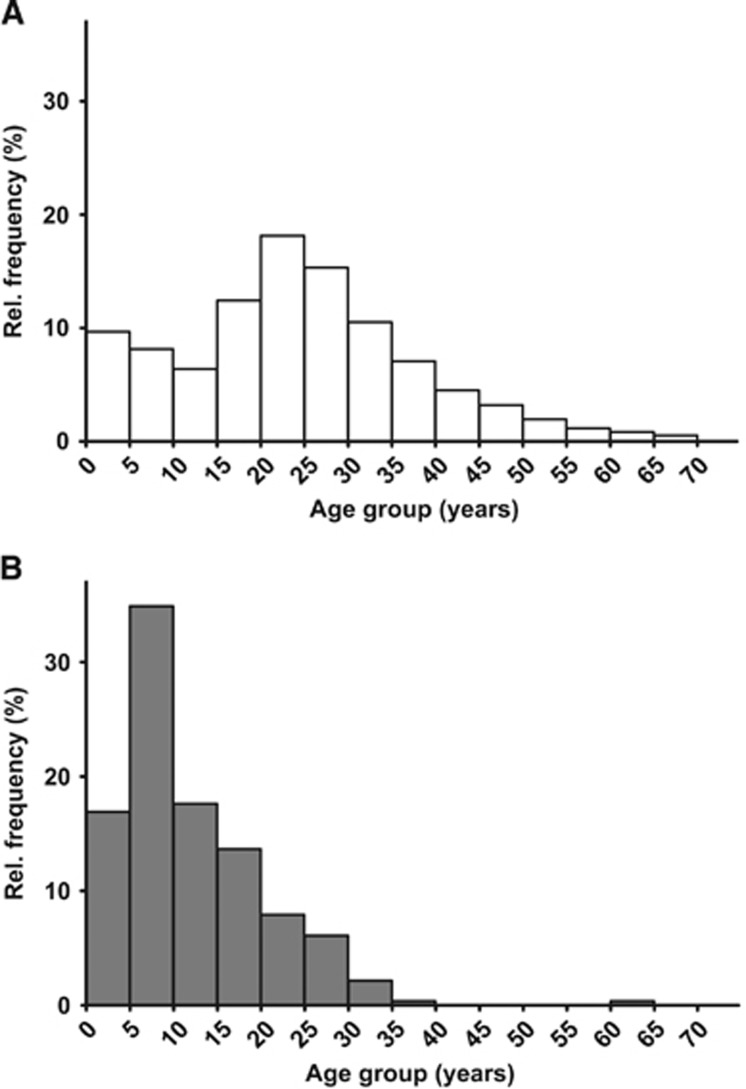
(**A**) Age distribution of arriving asylum seekers, Germany, September 2015 through March 2016. (**B**) Age distribution of notified hepatitis A cases in asylum seekers, Germany, September 2015 through March 2016.

Between September 2015 and March 2016, particularly young adults fled to Germany and are thus overrepresented among arriving asylum seekers. In this specific background population, asylum-seeking children and teenagers aged 0–19 years accounted for 37%, while children aged 5–9 years for 8% of all arriving asylum seekers (Figure 4A). Among all 278 hepatitis A cases in asylum seekers notified from September 2015 to March 2016 in Germany, children and teenagers aged 0–19 years accounted for 83% (231 cases), while children aged 5–9 years for 35% (97 cases; Figure 4B).

### Notified hepatitis A outbreaks

In total, 192 cases of the 278 cases in asylum seekers notified between September 2015 and March 2016 were linked to outbreaks of hepatitis A. Overall, 78 outbreaks were notified in this period, 50 (64%) of these outbreaks were related to HAV transmissions in asylum seekers, and occurred mainly in mass accommodations. Cluster sizes of hepatitis A outbreaks that were related to asylum seekers included up to eight cases with a median of two cases (IQR: 2–3 cases).

In the reference period, from September 2014 to March 2015, hepatitis A outbreaks in Germany totalled 44. Although the number of hepatitis A outbreaks almost doubled (increase of 77%), the cluster sizes did not significantly differ (*P*=0.33, Kruskal–Wallis test) from the respective five preceding periods (September to March in 2010/2011 to 2014/2015).

With the exception of a singular transmission from an asymptomatic child to a nursery nurse working in a mass accommodation, we are unaware of transmissions of HAV between asylum seekers and the German residential population.

### Molecular analysis of HAV strains in hepatitis A clusters

**Figure 5:**
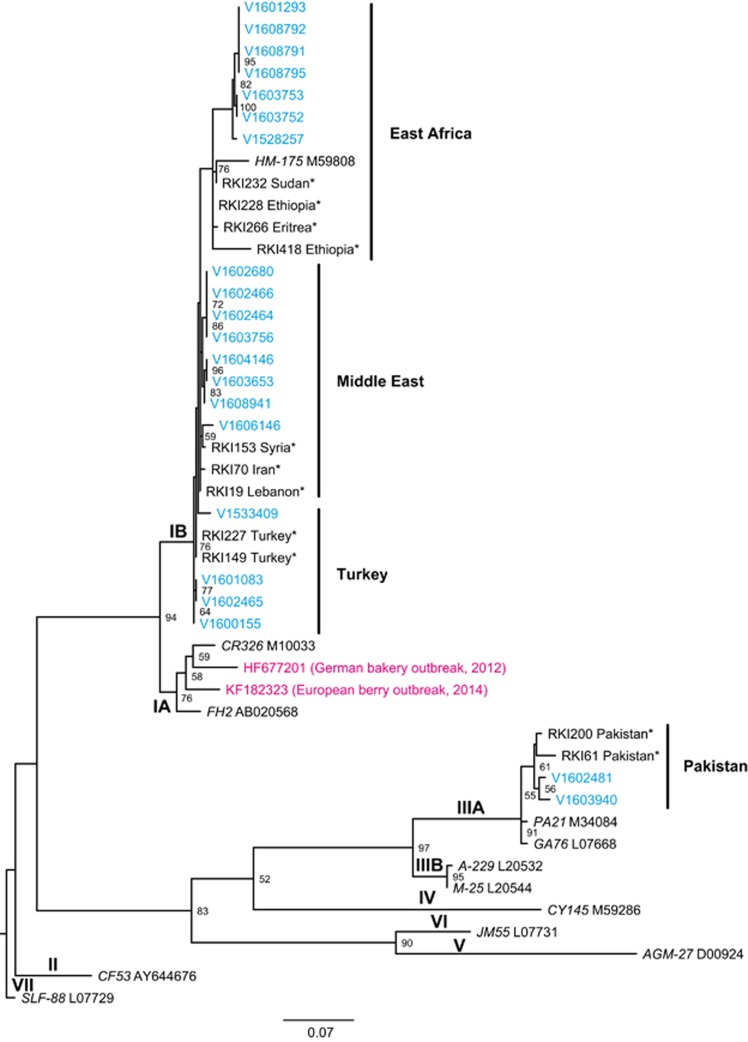
Phylogenetic tree of HAV samples (asylum seekers, Germany, September 2015 to March 2016) analysed by molecular typing of the VP1/P2A region. The VP1/P2A sequences of the strains related to asylum seekers (blue) cluster in HAV subgenotype IB (90%) and IIIA (10%). Archived sequences with known origin are marked with asterisks and depict four different geographical clusters (bold).^16^ Typical reference members of genotype I–VII are denoted by isolate name (italic) and GenBank ID.^17^ Genotype VII was used as an outgroup. Numbers at the nodes indicate bootstrap values of >50%. Two subgenotype IA sequences from recent European hepatitis A outbreaks are shown in magenta. The scale bar represents 0.07 substitutions per site.

Samples of stool, blood or serum from 21 hepatitis A cases in asylum seekers were sent for molecular sequence typing to the national consultant laboratory for HAV. Among the analysed samples 19 (90%) of the VP1/2A sequences were closely related and subgenotype IB, while two (10%) were subgenotype IIIA. Overall, we observed four clusters of nearly identical IB and IIIA sequences among the analysed HAV strains. The molecular signature of the sequenced samples related to HAV strains previously isolated from samples in the Middle East, Turkey, Pakistan and East Africa.^16^ The phylogenetic relations of all analysed HAV samples of arriving asylum seekers together with the previously analysed sequences of HAV reference strains are depicted in Figure 5.

## DISCUSSION

In 2015, the so-called refugee crisis put strong pressure on the European public health systems.^18^ In Germany, the number of asylum seekers peaked in the second half of 2015, with more than 800 000 people arriving.^19^ Due to the different geographical origin and the very often extremely challenging travel conditions to and in Europe, migrants are vulnerable to different infectious diseases than the ones affecting most of the EU resident population.^9, 18^

In this paper, we report an increase of hepatitis A notifications in Germany from September 2015 to March 2016 in temporal connection to the marked influx of asylum seekers. We hypothesized that a large proportion of the observed increase in hepatitis A notifications in Germany can be—at least partially—attributed to the 278 cases in asylum seekers. We demonstrate that the increase in hepatitis A notifications in 2015/2016 compared to 2014/2015 is of the same magnitude as the number of hepatitis A cases notified for arriving asylum seekers in this period (217 vs 278 cases) and, thus, is able to explain the increase of hepatitis A notifications in Germany.

However, the differentiation of the notified hepatitis A cases into residents and asylum seekers in Germany was only feasible after the introduction of additional variables into the national surveillance system in September 2015 implemented in reaction to the strong influx of asylum seekers in the second half of 2015. Therefore, no data on the residential status (asylum seeker or German resident) of hepatitis A cases before September 2015 is available as baseline information. We assume that the differentiation into residents and asylum seekers was still incomplete and would be primarily biased by unclassified asylum seekers in the notification system and, therefore resulted in an underestimation of hepatitis A cases in asylum seekers.

The increase of hepatitis A notifications in Germany reflects that arriving asylum seekers are largely exposed to HAV due to the fact that they predominantly originated from HAV endemic areas, for example, Middle East or Africa and, moreover experienced difficult hygienic and living conditions while migrating to Germany. Importantly, the level of HAV endemicitiy in the country of origin affects the level of naturally acquired immunity to HAV of children and adults in particular. Thus, WHO differentiates endemic areas into low, intermediate and high.^20^ In Germany, the incoming populations of asylum seekers originated mainly from intermediate (Middle East), low to intermediate (Albania and Serbia) but also from regions with high HAV endemicity (East Africa). In our study however, we could not differentiate hepatitis A cases in asylum seekers depending on their region of origin. Nevertheless, the reporting incidence ratio of 63 for hepatitis A when comparing asylum seekers as against the German general population underscores the comparatively high reporting incidence among this group. Of note, our calculation of the reporting incidence of hepatitis A in asylum seekers and the respective reporting incidence ratio referred to officially registered arrivals of asylum seekers who intended to apply for asylum in Germany (denominator) captured by the German EASY system.^2, 19^ We are aware that the exact number of arriving asylum seekers is hard to establish and the official figures might be subject to bias. However, this denominator—although it might be biased by misregistered or double-counted but also unregistered asylum seekers—should give at least a rough estimate of the arriving population and of the resulting reporting incidence of hepatitis A among asylum seekers.

Because of the aforementioned high exposure to HAV before coming to Germany, a large proportion of HAV infections in asylum seekers could be regarded as imported. Indeed, most hepatitis A cases in arriving asylum seekers occurred within a few days after arrival. However, taking into account arrival and disease onset dates and hepatitis A incubation time, as many as 24%–47% of HAV infections in asylum seekers are HAV infections apparently acquired in mass accommodations after arrival in Germany. The range of 24%–47% reflects the range of the incubation period of hepatitis A cases: 24% are very likely and further 23% were most likely acquired in Germany, pointing to a relatively high rate of ongoing transmissions within the group of asylum seekers after arrival to Germany.

By sequence typing of HAV samples from hepatitis A cases in arriving asylum seekers and phylogenetic analysis of the sequences, we could demonstrate that the samples are closely related to strains previously isolated from samples in the Middle East, Turkey, Pakistan and East Africa. These regions are known for endemic HAV circulation and are either starting points or located on the transit routes used by asylum seekers that fled to Europe in 2015/2016. Interestingly, 90% of the VP1/2A sequences we investigated are closely related and belong to subgenotype IB, while 10% were subgenotype IIIA. This is in contrast to the large food-associated hepatitis A outbreaks during the past years, where subgenotype IA was prevalent.^21, 22^ In our study, we observed several clusters of identical HAV sequences that suggested transmission among asylum seekers. Although we cannot completely exclude common sources of infection before asylum seekers arrived to Germany, these clusters might represent transmissions after arrival within the housing facilities for asylum seekers.

In the investigated period between September 2015 and March 2016, we noted a sharp decline in the median age of notified hepatitis A cases in Germany compared to the respective periods of previous years. In our report, we demonstrate that the decline is largely attributable to hepatitis A cases in asylum-seeking children. The age distribution of hepatitis A cases in the residential population remained unchanged as compared to previous years. Of note, our case definition does not include hepatitis A infections without symptoms, which are frequently observed in children. Assuming comparable infection risks for asylum seeking children aged one to four years compared to asylum seeking children aged five to nine years, the much lower reporting incidence observed in the former-mentioned group may indicate that circulation of HAV especially in this group is largely underestimated (Figure 4).

The observed high number of hepatitis A cases in asylum-seeking children and adolescents was not explainable by the unique age distribution of the arriving population. Comparing the age distributions of arriving asylum seekers and notified cases of hepatitis A in this group, reveals a strong relative overrepresentation of hepatitis A cases in asylum-seeking children and adolescents. This is in line with the observation that adult asylum seekers from countries where HAV is intermediately to highly endemic, such as Syria, might have acquired already a natural immunity to HAV.^20^ In addition, it mirrors the different vaccination recommendations of the WHO for hepatitis A, where regions are classified into low, intermediate and high HAV circulation. Overall, our data thus reveal the high level of exposure of asylum seekers to HAV, the occurrence of HAV transmission in mass accommodations, and, thus, suggest ongoing HAV circulation within this group after arrival in Germany. Specifically, we demonstrate that asylum-seeking children and adolescents are predominantly affected, both for imported and also autochthonous HAV infections.

As HAV infection and also vaccination confers immunity to HAV for decades, we suggest serological testing should be offered free-of-charge to clarify if immunity to HAV is already present. Essentially, hepatitis A virus immunization of asylum-seeking children and adolescents needs to be considered, although practical (vaccination schedule) and economical aspects may hinder the introduction of a general vaccination program. This would particularly prevent hidden infection chains and virus circulation that might possibly occur in connection to the admission of asylum-seeking children and adolescents into schools or day-care facilities, and impede HAV transmissions of asymptomatic cases into the residential population. The available hepatitis A virus vaccines are safe and highly effective and employed for routine childhood vaccination programs in some parts of the world.^23, 24, 25, 26, 27^ On that note, a single-dose hepatitis A vaccine was previously reported to be sufficient to confer long-lasting immunity to HAV.^28^ Furthermore, information campaigns could reinforce the awareness of infection risks and instruct on the prevention of HAV transmission. Vaccination and instructions on the prevention of HAV transmission should primarily target asylum seekers but also nursery nurses, teachers, assistants and staff in mass accommodations. In this context, the Standing Committee on Vaccination in Germany recommends vaccination against hepatitis A viruses for occupational indication for all volunteers or employees and others that are taking care for asylum seekers.

Moreover, we hypothesize that HAV transmission can be reduced if the housing conditions improve during the process of integrating asylum seekers into the residential population. Because crowding, poor sanitation and inadequate hygiene are well-established factors that are causative for faecal–oral transmission of HAV and particularly housing conditions in mass accommodations seem to be suboptimal to interrupt HAV transmission chains.

We report a substantial number of hepatitis A outbreak notifications from September 2015 to March 2016 in relation to asylum seekers admitted in mass accommodations. Apparently, regardless of strong efforts to accommodate asylum seekers under suitable living conditions, hepatitis A virus transmission was not completely avoidable. Nevertheless, cluster sizes of up to eight cases with a median of two cases do not significantly differ compared to previous years and point to effective control measures within the German public health system. Once hepatitis A cases were ascertained, extended hygiene measures, case isolation and post-exposure prophylaxis have been exerted to prevent secondary hepatitis A virus transmission.^29, 30^ Hence, local health departments have to remain vigilant to interrupt HAV transmission chains as early as possible to prevent further spread. In the investigated period, between September 2015 and March 2016, only a singular report of HAV transmission into the general population was reported to the Robert Koch Institute. Thus, spread of HAV seems to have been restricted to transmission within the group of asylum seekers and transmission to the German residential population was almost completely avoided highlighting the importance of rigorous hepatitis A surveillance and case management activities by public health authorities.

On that note, after the period of September 2015 to March 2016, hepatitis A notifications in Germany declined to levels comparable to previous years. We assume that in addition to successful public health measures also declining numbers of arriving asylum seekers in the first half of 2016 contributed markedly to reduced hepatitis A notifications in Germany. Thus, the observed increase of hepatitis A notifications in the last months seems to be entirely attributable to the reception of large numbers of refugees in the European Union.

In our study, the outlined increase of 45% in hepatitis A notifications between September 2015 and March 2016 in Germany refers to the preceding reference period of September 2014 to March 2015. To consider possible fluctuations of hepatitis A case counts in the surveillance system, we also included the median value of the respective five preceding time intervals that was nearly of the same magnitude and resulted in an increase of 42%. Overall, the increase of hepatitis A notifications in Germany was observed after an almost steadily decreasing trend in the last decade.

In summary, our report underlines that HAV transmission among asylum seekers is a relevant issue, particularly in asylum-seeking children and adolescents, but can be effectively mitigated by rigorous hygiene measures, case isolation and post-exposure prophylaxis. The high reporting incidence of hepatitis A in asylum seekers, that is largely limited to children, reflects the endemicity of HAV in the countries of origin and, additionally, the high exposure risk related to poor hygienic standards when asylum seekers flee to Europe. A large proportion of adult asylum seekers that originated from regions with intermediate or high HAV endemicity however, were largely exposed to HAV prior escaping their country of origin and acquired a natural immunity to HAV.^31^ Secondly, outbreaks of hepatitis A in mass accommodations were effectively controlled and of limited size, also the risk of HAV transmission into the residential population remained low. We are confident that the high vulnerability for migration-related sources of infections like HAV decreases as integration progresses. To this end, living conditions of asylum seekers ought to improve and converge towards those of the general population.
